# A Mutant with Expression Deletion of Gene *Sec*-1 in a 1RS.1BL Line and Its Effect on Production Quality of Wheat

**DOI:** 10.1371/journal.pone.0146943

**Published:** 2016-01-14

**Authors:** Zhi Li, Tianheng Ren, Benju Yan, Feiquan Tan, Manyu Yang, Zhenglong Ren

**Affiliations:** Agronomy College, Sichuan Agricultural University, Wenjiang, Chengdu, Sichuan, China; Institute of Genetics and Developmental Biology, CHINA

## Abstract

The chromosome arm 1RS of rye (*Secale cereal* L.) has been used worldwide as a source of genes for agronomic and resistant improvement. However, the 1RS arm in wheat has end-use quality defects that are partially attributable to the presence of ω-secalins, which are encoded by genes at the *Sec*-1 locus. Various attempts in removing the *Sec*-1 genes from the 1RS.1BL translocation chromosome have been made. In the present study, two new primary 1RS.1BL translocation lines, T917-26 and T917-15, were developed from a cross between wheat variety “A42912” and Chinese local rye “Weining.” The lines T917-15 and T917-26 carried a pair of intact and homogeneous 1RS.1BL chromosomes. The line T917-26 also harbored an expression deletion of some genes at the *Sec*-1 locus, which originated from a mutation that occurred simultaneously with wheat-rye chromosome translocations. These results suggest that the accompanying mutations of the evolutionarily significant translocations are remarkable resources for plant improvement. Comparison of translocation lines with its wheat parent showed improvements in the end-use quality parameters, which included protein content (PC), water absorption (WA), sodium dodecyl sulfate sedimentation (SDSS), wet gluten (WG), dry gluten (DG) and dough stickiness (DS), whereas significant reduction in gluten index (GI) and stability time (ST) were observed. These findings indicate that 1RS in wheat has produced a higher amount of protein, although these comprised worse compositions. However, in the T917-26 line that harbored an expression deletion mutation in the *Sec*-1 genes, the quality parameters were markedly improved relative to its sister line, T917-15, especially for GI and DS (P < 0.05). These results indicated that expression deletion of *Sec*-1 genes significantly improves the end-use quality of wheat cultivars harboring the 1RS.1BL translocation. Strategies to remove the *Sec*-1 genes from the 1RS.1BL translocation in wheat improvement are discussed.

## Introduction

Rye (*Secale cereale* L.) long has been recognized as valuable sources of genes for wheat improvement [1], and the short arm of rye chromosome 1R via the 1RS.1BL and 1RS.1AL translocations now resides within the genome of a large number of wheat breeding lines and elite cultivars [2,3,4,5,6,7]. Rabinovich [8] listed around 300 cultivars that harbor the 1RS chromosome arm. From the 1980s until the 2000s, approximately 70% of wheat cultivars released in southwestern China and 55% in northern China carry this chromosome arm [9, 10]. The widespread distribution of 1RS around the world has provided advantages in wheat improvement programs. A number of resistant genes of rye (*Secale cereale* L.) are located in chromosome arm 1RS such as genes for resistance to different rust pathogens (*Lr26*, *Sr31*, *Yr9*, and *YrCn17*), powdery mildew (*Pm8* and *PmCn17*) [11, 12, 13, 14, 15], greenbug (*Gb2*) [16, 17, 18], and the Russian wheat aphid (*Dn7*) [19, 20]. In addition to disease and pest resistance, the 1RS arm in wheat-rye translocation also confers higher yield and adaptive advantages to wheat in various environments [5, 21, 22, 23, 24]. Comparison of the genotypes carrying the 1RS.1BL translocation with those harboring its homologous 1B chromosomes revealed that 1RS.1BL lines have higher grain and aboveground biomass yield, increased kernel weight, and better post-anthesis stress tolerance [25, 26, 27]. Furthermore, Howell et al. [28] discovered a small distal region in the 1RS arm of the 1RS.1BL translocation that has a positive impact on wheat yield and canopy water status. Ren et al. [29] identified a gene(s) in the 1RS chromosome arm that generates a stay green trait at the later filling stage that interacts with the wheat background. Based on these findings, it is thus expected that the 1RS.1BL translocation will continue to impact breeding programs around the world [30].

Unfortunately, 1RS also is known to have detrimental effects on grain processing quality [31, 32, 33]. The most typical defects include the production of sticky dough, poor fermentation, inferior dough-mixing properties, and low SDS sedimentation volumes [34, 35]. These undesirable dough properties are considered to be partially caused by the presence of ω-secalins, which are a family of small monomeric proteins, with a molecular range (Mr) of 45–55 kD, and encoded by the genes at the *Sec*-1 locus on the 1RS arm [36,37,38].

Several strategies have been utilized to eradicate the deleterious quality effects of the genes at the locus *Sec*-1 on the 1RS.1BL translocation. It is quite clear that the magnitude of the effect of 1RS on quality varies with genetic background [23, 39, 40]. Significant differences among primary 1RS.1BL lines that originated from different wheat and rye parents have been observed [27]. These primary translocation lines would have the best chances to produce 1RS.1BL lines with acceptable dough strength. Chromosome engineering by induced homoeologous recombination could also be exploited to remove the associated breadmaking quality defect of the 1RS.1BL translocation [41, 42]. Another approach is to use RNA interference methods to silence the expression of target genes [43]. However, *Sec*-1, which is located on the 1RS arm, is a complex locus [44, 45], and more attempts for removing these genes from this particular arm would be necessary.

In the present paper, we report a new primary 1RS.1BL translocation line that we have determined to be an expression deletion mutant of some genes at the *Sec-*1 locus.

## Materials and Methods

### Plant material

A common wheat (*Triticum aestivum* L.) genotype, A42912, was crossed with a Chinese local rye (*S*. *cereal* L.) variety, Weining. The F1 seedlings were soaked in 0.05% colchicine + 3% dimethyl sulfoxide for 8 h to produce the amphidiploid (C1). The C1 plants were backcrossed once or twice using the parental wheat variety A42912 to produce 1R monosomic wheat/rye addition lines. The 1R monosomic addition lines were then propagated by selfing in the isolation field. From the progeny population of monosomic addition lines, the primary 1RS.1BL translocation lines were selected [46, 47, 48]. Weining rye was collected from southwestern China. Wheat variety A42912 is a pure line of the common wheat that harbors the *kr2* gene and can easily be crossed with rye. No rye chromatin in the genome of A42912 was detected by using the genome in situ hybridization (GISH) technique. A42912 is highly susceptible to stripe rust (caused by *Puccinia striiformis* f. sp. *tritici*, Pst) and powdery mildew (caused by *Blumeria graminis* f. sp. *tritici*, Bgt) in China. However, the A42912-1R monosomic addition line is resistant to these two diseases. Seeds of A42912 used in the present study were produced by single spike descent across several generations to create pure genetic stocks. Wheat cultivars Chuan-Nong 10 (CN10), which inherited their 1RS.1BL translocation chromosome from the Russian wheat cultivar Aurora, and Chuan-Nong 17 (CN17), of which the 1RS arm was derived from rye inbred line L155 (from Hohenheim, Germany), were used as controls. CN10 and CN17 were released in 2000 and 2003, respectively, and distributed across Southwest China.

### Selection of translocation lines and identification of translocation chromosomes

The root mitotic metaphase cells of each plant from the progeny population of monosomic addition lines were prepared according to the methods described by Ren [48] and Han [49]. Fluorescence in situ hybridization (FISH) and GISH were used to identify the mitotic metaphase cells. The genomic DNA of Weining rye, the *Aegilops tauschii* clone pAs1, and the rye clone pSc119.2 were used as probes to identify the chromosomes of wheat and rye [50, 51]. The clones pAWRC.1, 6c6, and sequence CCCTAAACCCTAAACCCTAAACCCTAAA were used as probes to identify the centromeres and telomeres, respectively [52, 53]. The genomic DNA of Weining rye and the repetitive sequences pAs1 and pAWRC.1 were labeled with Texas Red-5-dUTP (Invitrogen). The repetitive sequences pSc119.2 and 6c6 and the telomere probe were labeled with Alexa Fluor-488-5-dUTP (Invitrogen). Probe labeling and *in situ* hybridization were also performed according to Han et al [49]. Images were captured using an epifluorescence microscope (BX51, Olympus) equipped with a cooled charge-coupled device camera, operated using an HCIMAGE Live software (version 2.0.1.5), and processed with Photoshop CS 3.0.

The plants that were determined to contain a translocation chromosome were further grown in an isolated field for the selection of pure lines of translocation. The translocation line, which contains a pair of homozygous translocation chromosomes and exhibits consistent main traits, was considered as a new primary translocation line.

### Electrophoretic detection of ω-secalin and gliadin proteins (A-PAGE)

A-PAGE was conducted according to the procedure of Payne et al. [54], with minor modifications. For the extraction of gliadin and ω-secalin proteins, the crushed seeds were incubated in 70% (v/v) ethanol (3.3 μL per mg grain) for 2–3 h at room temperature with vortex mixing. The suspension was then centrifuged at 8,000g for 5 min in a microfuge. Then, the supernatant was mixed with 0.84 of its volume of 60% (v/v) glycerol and 0.05% (w/v) pyronin G, and the sample was centrifuged again to collect the sediment. Approximately 50 μL of the supernatant was used for electrophoresis. Samples were loaded onto 2 mm-thick 6% acrylamide gels and 0.3% (w/v) methylenebisacrylamide buffered at pH 3.1. The proteins were fractionated at a constant voltage of 180V for approximately 60 min until the tracking dye, pyronin G, ran off the gel. The gels were stained in 12% trichloroacetic acid (TCA) with 0.04% Coomassie brilliant blue G-250 and destained in 12% TCA [13, 38, 55, 56].

### Evaluations of grain and flour quality

All 1RS.1BL translocation lines and their parents were planted with three replications in the field in Qionglai District (30°25′N, 103°28′E, and 493.3 m above sea level), Chengdu, Sichuan, China between 2012 and 2014. Each plot was 2 m long and 25 cm apart, and contained four rows with 80 seeds in each row. Field management followed standard agricultural practices. After harvesting grain samples of the new 1RS.1BL lines, its wheat parent and controls were analyzed in the laboratory. Evaluation of grain and flour quality was performed according to the procedures described by Kumlay et al. [24], Burnett et al. [34], Carver and Rayburn [35], Martin and Stewart [57], and Martín et al. [58], with minor modifications. Whole wheat was milled with a 0.5-mm screen (FOSS 1093 Cyclotec Sample Mill, Sweden). The flour was milled using a Laboratory Mill (Brabender Measurement and Control Systems, Germany). The wet gluten content (WG) and gluten index (GI) were examined with a Glutenmatic 2200 (Perten, Sweden). Protein content (PC) was detected by using a distillation unit B-324 (Buchi, Sweden) [59]. Sodium dodecyl sulfate sedimentation (SDSS) was measured according to Dexter et al. [60]. Dough stickiness was assessed following a procedure by Burnett et al. [35] and Dhaliwal and MacRitchie [36].

### Statistical analysis

ANOVA was performed on the collected data on each quality parameter. The respective error term for the F-test was estimated using PROC GLM from PC-SAS 9.1. Least significant differences (LSD) were calculated for mean comparisons.

## Results

### Development of the new primary 1BL.1RS translocation lines

**Fig 1:**
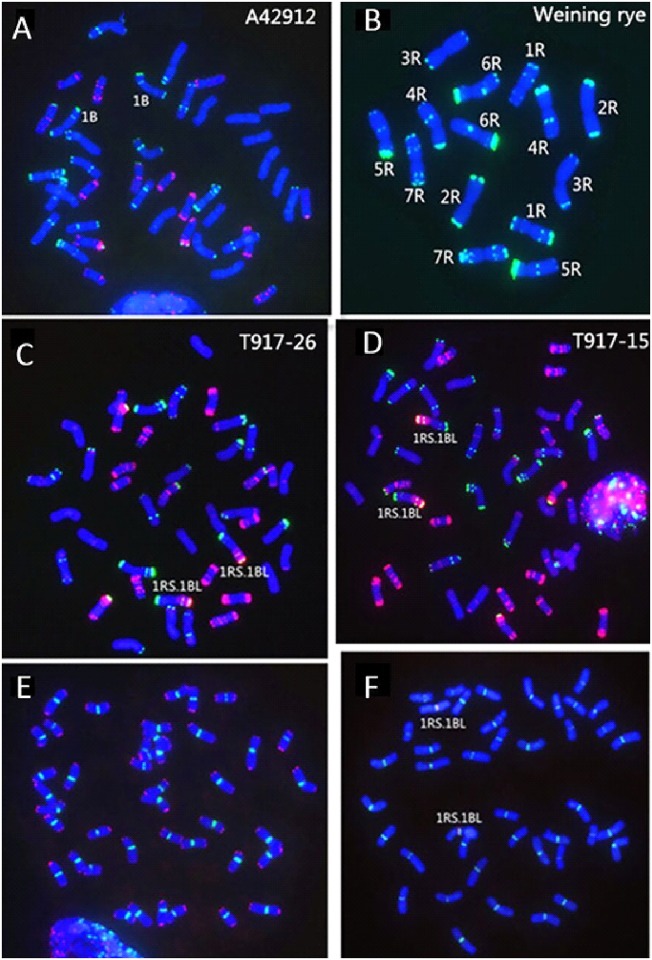
Identification of translocation chromosomes. (A)–(D), FISH and GISH using rye genomic DNA (red), pSc119.2(green), and pAs1 (red) as probes. (A) and (B), Wheat and rye parents. (C) and (D), new primary 1RS.1BL translocation lines. (E), FISH using 6c6 (green) and telomere sequence (red) as probes, indicating the integrity of all chromosomes. (F), FISH using 6c6 (green) and pAWRC.1 (red) as probes, indicating that the 1RS arm is intact in the translocation.

The wheat variety A42912, which harbored a pair of intact 1B chromosomes (Fig 1A), was easily crossed with rye variety Weining (Fig 1B). The amphidiploid plants (C_1_) were backcrossed twice with the wheat parent. From the progenies of hybrids (BC_2_F_1_), a monosomic addition plant of 1R (2n = 43) was selected, which exhibited a high level of resistance to stripe rust and powdery mildew and was designated as line 98–917. The progenies of the 98–917 plant were grown in an isolation field. The progeny plants showing resistance to strip rust and powdery mildew were sequentially selected and reproduced. In the BC_2_F_5_ population, five plants were selected and identified by means of FISH and GISH techniques to harbor a pair of 1RS.1BL translocation chromosomes (Fig 1C and 1D). No major differences among these translocation chromosomes of the five plants were observed using the FISH and GISH techniques (Fig 1). FISH analysis using clone 6c6 as a centromeric probe and a repetitive sequence as telomeric probe demonstrated that all these plants carried 42 intact chromosomes (Fig 1E). FISH using clones of 6c6 and species-specific probe of centromere of rye, pAWRC.1, showed that an intact 1RS chromosome arm existed in the 1RS.1BL translocation. The five plants were further reproduced as lines, respectively. Similar morphological traits for plant height, spike, leaves, seeds, and growth habit were observed among the five primary translocation lines. Compared to their wheat parent, these translocation lines showed better agronomic traits and were highly resistant to strip rust and powdery mildew in the field.

### Identification of expression deletion of *ω-secalins*

**Fig 2:**
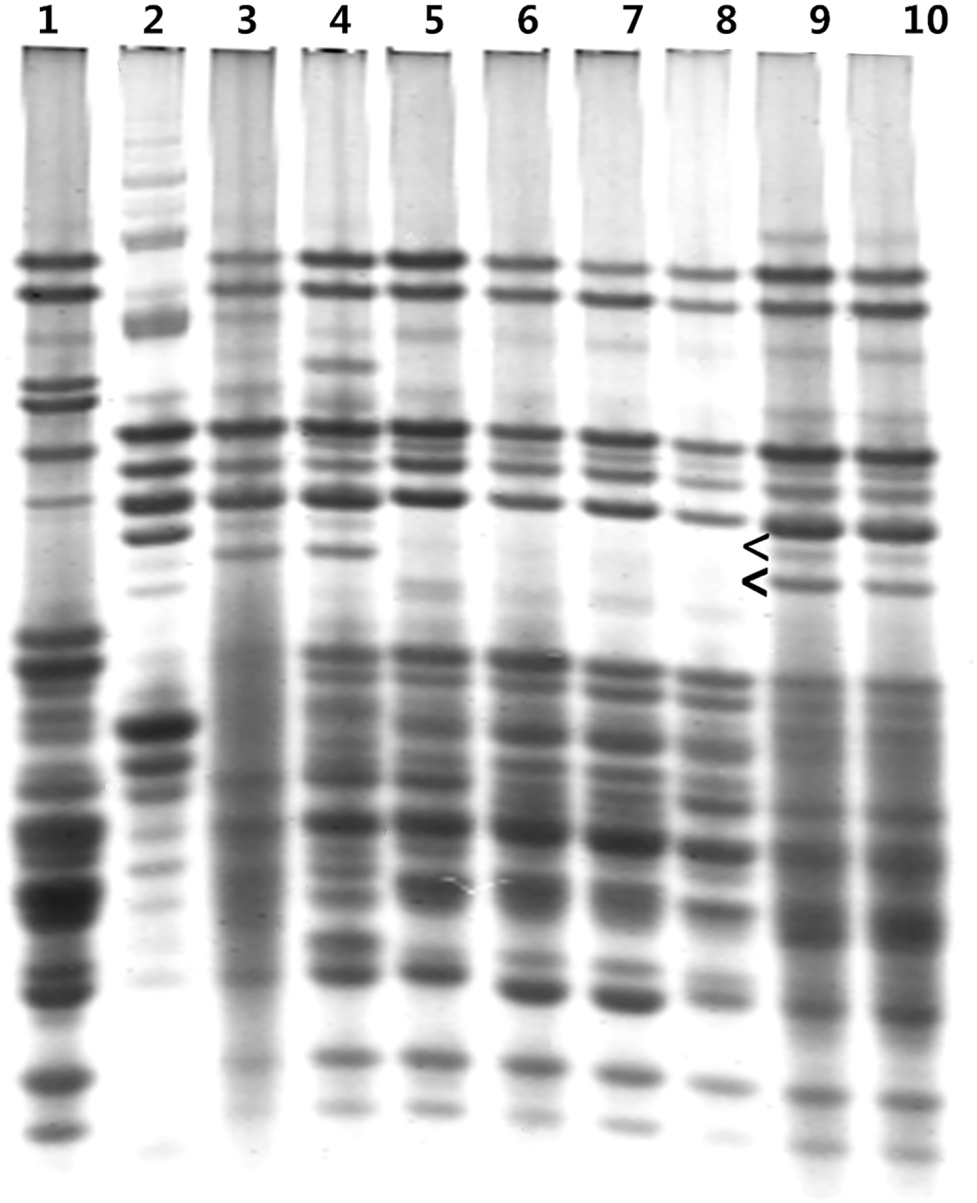
A-PAGE separations of ω-secalins and gliadins from a new primary 1RS.1BL translocation line and its wheat and rye parents. (L to R) 1 = wheat parent A42912, 2 = Weining rye, 3 = Chuan-nong 10, 4 = Chuan-nong 17, 5–8 = different lines of T917-26, the seeds from two years, 9 and 10 = different lines of T917-15. Arrowheads indicate the absence of expression of ω-secalins.

The expression of the genes at the *sec*-1 locus in the five primary 1RS.1BL translocation lines was investigated by A-PAGE. The three lines exhibited normal expression for the genes at the *Sec*-1 locus, whereas two lines showed an absence of expression of some genes at the *Sec*-1 locus (Fig 2). Because these primary 1RS.1BL translocation lines originated from the same plant with a 1R monosomic addition (2n = 43), these exhibited similar morphological characteristics. The two lines that did not express some genes at the *Sec*-1 locus were named T917-26, whereas the other three lines that showed intact expression of genes at the *Sec*-1 locus are designated as T917-15. Fig 2 shows the consistent observation that some genes at the *Sec*-1 locus were not expressed in the seeds harvested after two years (lanes 5 and 6, and 7 and 8), which indicated that the expression deletion mutation of the genes at *Sec*-1 locus in 1RS.1BL translocation line T917-26 was stable.

### Effects of expression deletion of wheat *Sec*-1 genes on grain processing quality

Significant differences in several traits (P < 0.05) between the primary 1RS.1BLtranslocation lines and its wheat parent were observed, which included WG, dry gluten (DG), SDSS, water absorption capacity (WA), and stability time (ST), and dough stickiness (DS), with the exception of development time (DT) (Table 1). The introduction of the 1RS arm from Weining rye into wheat line A42912 resulted in a marked increase in PC, WG and DG content, SDSS, and WA, but a remarkable decrease for GI and ST, and a trend of greater DS. These alterations in 1RS.1BL translocation lines may actually lead to deleterious effects on various end-use quality traits. However, comparison of the 1RS.1BL translocation line T917-15 with its sister line T917-26 showed that the expression deletion of some genes at the *Sec*-1 locus on the 1RS arm notably improved its quality traits (Table 1).

**Table 1:** Effects of expression deletion mutation of *Sec*-1 genes in primary 1RS.1BL lines on the quality traits.

	PC	WA	SDSS	WG	DG	GI	DT	ST	DS
A42912	13.18ab	60.6b	44.0ab	36.41bc	12.19ab	89.27a	4.22b	8.82a	7.7c
T917-26	14.11a	61.9ab	54.4a	39.23ab	13.39a	67.15b	4.27b	6.44ab	12.7b
T917-15	14.67a	62.1a	47.9ab	42.27a	13.79a	42.45c	3.30b	4.58b	18.3a
CN10	12.46b	61.8ab	27.2b	31.98c	10.25b	58.91bc	4.19b	3.21b	9.3bc
CN17	14.35a	61.6ab	44.8ab	37.42bc	12.63ab	81.96a	6.91a	7.43a	18.7a

PC, protein content; WA, water absorption; SDSS, sodium dodecyl sulfate sedimentation; WG, wet gluten; DG, dry gluten; GI, gluten index; DT, development time; ST, stability time; DS, dough stickiness. Values with the same letter in the same column do not differ significantly at P< 0.05.

## Discussion

### Alleviating deleterious effects of 1RS.1BL translocation on end-use quality traits

Wheat bread-making quality is influenced by a complex collection of various factors such as genetic background, environmental conditions, and agricultural practice, which function mainly by controlling grain protein concentration and compositions [61]. The effects of the 1RS chromosome arm on PC in 1RS.1BL translocation lines are very diverse, and depend on the origin of rye and its interaction with a wheat genetic background [27]. In the present study, comparison of translocation line T917-15 with its wheat parent A42912 showed that genetic difference only existed between the 1RS and 1BS arms. A significantly higher PC was observed in the new primary 1RS.1BL translocation line T917-15 compared to its wheat parent A42912 (Table 1), which was also accompanied by an increase in WG, DG, SDSS, WA and DS. However, the remarkable decrease in GI and ST simultaneously occurred, which are the main wheat quality parameters in the commercial market [62]. The decrease in GI and ST generally leads to low flour quality. These results suggest that the introduction of the 1RS arm into wheat resulted in higher protein yield, although the composition of proteins encoded by the genes on the 1RS arm was of an inferior quality to those of the 1BS arm. These undesirable protein properties are believed to be partly caused by the production of ω-secalins, which are a family of small monomeric proteins with a Mr of 45–55 kd. They replaced the ω-gliadins that are encoded by wheat genes on the 1BS arm [36]. Several strategies have been exploited to minimize the deleterious effects of the 1RS.1BL translocation on the composition of proteins, including the utilization of genetic diversity [23, 27, 39, 40], chromosome engineering by induced homoeologous recombination [41, 42], and directly removal of the *Sec*-1 genes by using RNA interference [43]. However, *Sec*-1 on the 1RS arm is a complex locus [44, 45], and thus, all attempts to remove the *Sec*-1 genes have got few results so far.

In the present study, we developed a new 1RS.1BL translocation line, T917-26, by crossing wheat with rye. The line T917-26 harbors an expression deletion mutation of some genes at the *Sec*-1 locus on the 1RS arm, which affects ω-secalins and in turn, the end-use quality traits of wheat.

### Effects of expression deletion of wheat *Sec*-1 genes on grain processing quality

The lines T917-26 and T917-15 were fully homogeneous. They have same genetic background of wheat and 1RS, in which T917-26 is different from T917-15 only in expression deletion of *Sec*-1 genes and some mutations of low-molecular prolamin. Notable differences between the two translocation lines were observed for PC, SDSS, WG, ST, and WA and DS (Table 1). The quality difference between them would originate from expression deletion of *Sec*-1 genes and from some low-molecular prolamin mutations. However, effect of *Sec*-1 genes on wheat quality is much more important than effect of low-molecular prolamin [63, 64]. This results indicating that the expression deletion of some genes at the *Sec*-1 locus has modified the compositions of wheat proteins. Significant differences in GI and DS (P < 0.05) were also observed as a consequence of the expression deletion of some genes at the *Sec*-1 locus. GI and DS is an important parameter for wheat quality in the commercial market [62]. These results indicated that attempts to remove the *Sec*-1 genes from 1RS.1BL translocation were successful.

### Origin of the expression deletion mutant of *Sec*-1 genes

Rye (*S*. *cereal* L.) is a cross-pollinating species consisting of genetically diverse populations with morphologically similar features. The presence of diverse genes in the 1RS arms from different plants of such populations is not unexpected. Because of genetic differences among 1RS arms and the interaction between genes on 1RS and wheat background, it is easy to understand that genetic diversity occurs in 1RS.1BL translocations derived from the same wheat parent and rye variety. However, in the present study, 1RS.1BL translocation lines T917-26 and T917-15 derived from the same 1R monosomic addition plant, which originated from a wheat pure line and one 1R chromosome. The T917-26 and T917-15 is thus assumed to contain identical wheat chromosomes and 1RS arm. The experimental results identified differences in the *Sec*-1 between these two sister lines. The expression deletion of the *Sec*-1 genes was a mutation that simultaneously occurred with the wheat-rye chromosome translocation. Frequent aberrations of wheat and rye chromosomes have been observed, which were induced by the monosomic addition of rye chromosomes into that of wheat [50]. The results of the present study described the effects of induction of evolutionarily significant translocations in wheat and rye chromosomes. The accompanying mutations may be utilized as valuable resources for plant improvement.

### Strategies to improve the quality of the wheat cultivars harboring the 1RS.1BL chromosome

Various methods have been utilized to improve the processing quality of 1RS.1BL wheat. The procedure to remove *Sec*-1 genes could directly improve the composition of wheat proteins. In the present study, we have developed a mutant that harbored expression deletion of some genes at the *Sec*-1 locus on the 1RS arm. The mutation simultaneously occurred with evolutionarily significant translocations between chromosomes of wheat and rye. Using this approach, different new 1RS.1BL lines may be developed, which could contain various expression deletion mutations of genes at the *Sec*-1 locus on the 1RS arm. Through genetic recombination of different expression deletion mutants of *Sec*-1 genes, we have developed new 1RS.1BL lines, which carried the 1RS.1BL chromosome with partial or whole gene deletions at the *Sec*-1 locus. Using a combination of suitable gluten in alleles and deletion mutants of *Sec*-1 genes, it is possible to improve the end-use quality of wheat cultivars that harbor the 1RS.1BL chromosome [33, 39, 65].

## Conclusions

The simultaneous occurrence of the evolutionarily significant translocation of the wheat-rye chromosomes also results in gene mutations, which we call accompanying mutations. The accompanying mutations are valuable resources for plant improvement. A mutant with the expression deletion of some genes at the *Sec*-1 locus on the 1RS arm was selected and identified by using FISH, GISH, and biochemical analyses. The new 1RS.1BL translocation line with an expression deletion mutation of *Sec*-1 genes possessed better end-use quality traits than its sister line, which did not carry this particular mutation. The disadvantageous genes in evolutionarily significant translocations may be effectively removed by using this new method.
